# Evaluation of four chimeric *Trypanosoma cruzi* recombinant antigens for serological diagnosis of chronic Chagas disease in dogs: a phase II study

**DOI:** 10.1186/s13071-025-07173-4

**Published:** 2025-12-07

**Authors:** Natália Dantas Fontes, Fernanda Lopes Habib, Leonardo Maia Leony, Natalia Erdens Maron Freitas, Ângelo Antônio Oliveira Silva, Filipe Dantas-Torres, Kamila Gaudêncio da Silva Sales, Andréa Pereira da Costa, Thaliane França Costa, Nayara Mendes Louzeiro, Sidilene Pereira Costa, Lileia Gonçalves Diotaiuti, Carlota Josefovcz Belisario, Cláudia Moura de Melo, Antônia Cláudia Jácome da Câmara, Vicente Toscano de Araújo-Neto, Luanna Soares de Melo Evangelista, Deborah Bittencourt Mothé, Paola Alejandra Fiorani Celedon, Nilson Ivo Tonin Zanchin, Fred Luciano Neves Santos

**Affiliations:** 1 Advanced Public Health Laboratory, Gonçalo Moniz Institute, Oswaldo Cruz Foundation, Salvador, Brazil; 2 Interdisciplinary Research Group in Biotechnology and Epidemiology of Infectious Diseases (GRUPIBE), Gonçalo Moniz Institute, Oswaldo Cruz Foundation (FIOCRUZ-BA), Salvador, Brazil; 3 Laboratory of Immunoparasitology, Department of Immunology, Aggeu Magalhães Institute, Oswaldo Cruz Foundation, Recife, Brazil; 4 Postgraduate Program in Animal Science, State University of Maranhão, São Luís, Brazil; 5 Triatomines and Epidemiology of Chagas Disease, René Rachou Institute, Oswaldo Cruz Foundation, Belo Horizonte, Brazil; 6 Integrated Translational Program in Chagas Disease from Fiocruz – Fio-Chagas, Rio de Janeiro, Brazil; 7 Postgraduate Program in Health and Environment, Tiradentes University, Aracaju, Brazil; 8 Institute of Technology and Research, Aracaju, Brazil; 9 Department of Clinical and Toxicological Analysis, Health Sciences Center, Federal University of Rio Grande Do Norte, Natal, Brazil; 10 Postgraduate Program in Pharmaceutical Sciences, Federal University of Rio Grande Do Norte, Natal, Brazil; 11 Department of Parasitology and Microbiology, Health Sciences Center, Federal University of Piauí, Teresina, Brazil; 12 Laboratory of Parasite-Host Interaction and Epidemiology, Gonçalo Moniz Institute, Oswaldo Cruz Foundation, Salvador, Brazil; 13 Laboratory for Applied Science and Technology in Health, Carlos Chagas Institute, Oswaldo Cruz Foundation, Curitiba, Brazil; 14 Laboratory of Structural Biology and Protein Engineering, Carlos Chagas Institute, Oswaldo Cruz Foundation, Curitiba, Brazil

**Keywords:** Chagas disease, *Trypanosoma cruzi*, Immunodiagnostics, Recombinant chimeric antigens, Dogs, Canine trypanosomiasis, Anthropozoonosis

## Abstract

**Background:**

Dogs are recognized as epidemiologically significant reservoirs for *Trypanosoma cruzi*, the causative agent of Chagas disease (CD), owing to their close association with humans and their role in sustaining the domestic and peridomestic transmission cycle. Canine seropositivity often correlates with human CD prevalence. However, the lack of commercial, high-performance diagnostic assays for canine infections remains a significant barrier to effective surveillance. Previously, our group demonstrated the diagnostic potential of four chimeric *T. cruzi* antigens in a phase I study, yielding results comparable to those observed in humans. The present phase II study expands upon these findings by evaluating these antigens in a larger canine cohort using indirect enzyme-linked immunosorbent assay (ELISA). The objective of this study was to assess the diagnostic performance of four chimeric recombinant *T. cruzi* antigens (IBMP-8.1, IBMP-8.2, IBMP-8.3, and IBMP-8.4) in immunoassays for the detection of anti-*T. cruzi* IgG in dogs with chronic Chagas disease.

**Methods:**

Immunoassays were optimized by checkerboard titration. In this phase II study, the diagnostic performance of the IBMP antigens was evaluated using 1260 canine serum samples. Cross-reactivity was assessed in an additional 752 samples from dogs with unrelated infectious diseases. The performance of the chimeric antigens was compared with a commercial human-adapted assay (Gold ELISA Chagas).

**Results:**

The Instituto de Biologia Molecular do Paraná (IBMP) antigens demonstrated area under the curve (AUC) values ranging from 89.0% to 97.4%, with diagnostic accuracy between 87.4% and 96%. IBMP-8.2 exhibited the highest sensitivity (90.3%), while IBMP-8.1, IBMP-8.3, and IBMP-8.4 achieved sensitivities of 74.8%, 72.6%, and 79.6%, respectively. The highest specificity was observed for IBMP-8.4 (99.6%), followed by IBMP-8.3 (99.0%), IBMP-8.2 (96.5%), and IBMP-8.1 (90.6%). The Gold ELISA Chagas assay showed a sensitivity of 62.3%, specificity of 98.6%, and accuracy of 89.9%. IBMP-8.2 exhibited the lowest cross-reactivity index (0.9%), closely approximating an ideal diagnostic assay.

**Conclusions:**

The IBMP chimeric antigens demonstrated strong diagnostic performance for detecting *T. cruzi* infection in dogs, significantly enhancing immunoassay accuracy and minimizing diagnostic failures due to cross-reactivity. The combined use of these antigens represents a promising strategy to further improve sensitivity and specificity in future diagnostic applications.

**Graphical Abstract:**

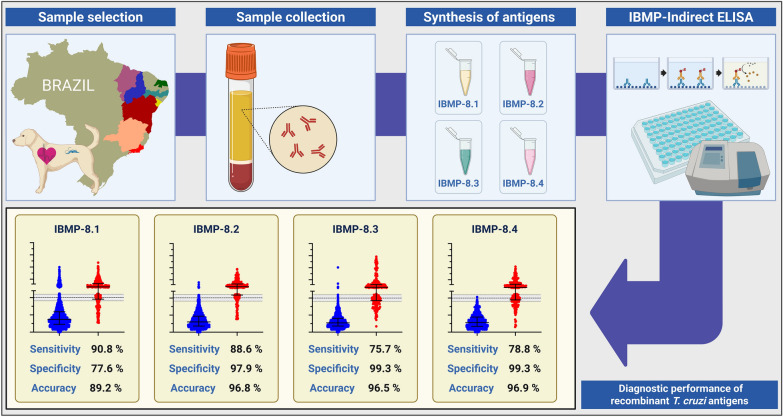

**Supplementary Information:**

The online version contains supplementary material available at 10.1186/s13071-025-07173-4.

## Background

Chagas disease (CD), caused by the protozoan parasite *Trypanosoma cruzi*, is a neglected tropical disease that poses a significant threat to both human and animal health. In Latin America, where CD is endemic, an estimated 7.5 million individuals are chronically infected [1], resulting in approximately 12,000 deaths annually [2]. The primary mode of transmission is vectorial, via the feces of infected triatomine bugs, placing nearly 100 million people at risk [1]. Additional transmission routes include blood transfusion, congenital infection, ingestion of contaminated food or beverages, and accidental laboratory exposure [3]. In recent decades, increased human migration has facilitated the global spread of *T. cruzi*, transforming CD into an emerging public health concern in nonendemic regions, such as Europe, North America, Asia, and Oceania [4, 5].

More than 100 mammalian species have been identified as natural hosts for *T. cruzi* [6], including domestic animals such as dogs, cats, and pigs. These animals attract triatomine vectors seeking blood meals and may become infected through vectorial, vertical, transfusional, or oral routes [7–10]. Among domestic animals, dogs are important as sentinels of *T. cruzi* transmission in endemic settings [7, 11]. Their serological status serves as an early indicator of parasite circulation and potential risk to nearby human populations [12]. Canine seroprevalence often correlates with transmission intensity, making dogs valuable sentinels for eco-epidemiological surveillance and targeted public health interventions. Furthermore, dogs can develop clinical manifestations and physiopathological changes similar to those observed in humans, making them patients for veterinary care and valuable experimental models for CD [13–15]. In severe cases, sudden death may occur [16]. Despite their diagnostic and epidemiological relevance, few commercial diagnostic tests are currently available for detecting CD in dogs.

Laboratory diagnosis of CD depends on the phase of infection. During the acute phase, which is typically asymptomatic and brief, circulating parasites can be detected by microscopic examination. In contrast, the chronic phase is characterized by low and intermittent parasitemia, requiring serological methods, such as indirect immunofluorescence, rapid diagnostic tests, and enzyme-linked immunosorbent assays (ELISA), to detect anti-*T. cruzi* antibodies. However, these assays are often hampered by factors that compromise their reliability, including the genetic and phenotypic diversity of the parasite [17], antigen selection [18], variation in disease prevalence [19, 20], host immune response heterogeneity [21], and cross-reactivity with *Leishmania* spp. [22, 23]. Consequently, the Pan American Health Organization (PAHO) recommends the use of two distinct serological assays for diagnosing CD in humans [24]. In contrast, CD in dogs still relies on in-house or adapted human serological tests [25–29], contributing to underreporting and delays in clinical confirmation.

To enhance the serodiagnosis of chronic CD, chimeric recombinant proteins comprising conserved and repetitive epitopes from multiple *T. cruzi* antigens have been developed for use in diagnostic platforms [30–33]. The Instituto de Biologia Molecular do Paraná (IBMP) chimeric proteins (IBMP-8.1, IBMP-8.2, IBMP-8.3, and IBMP-8.4) have demonstrated high diagnostic accuracy in human studies across South America [23, 26, 34–41], as well as in nonendemic regions such as Barcelona, Spain [42]. These antigens have also been explored as candidates for human vaccine development [43] and have recently shown promising results in a phase I diagnostic study involving naturally and experimentally infected dogs from various regions of Brazil [25–27, 44, 45]. Building upon these findings, the present phase II study aims to evaluate the diagnostic performance of IBMP chimeric antigens for detecting anti-*T. cruzi* antibodies in canine sera from endemic regions across Brazil.

## Methods

### Synthesis of chimeric antigens

The chimeric antigens employed in this study were synthesized as previously described by Santos et al. [34]. Briefly, gene sequences encoding the chimeric proteins were cloned into the pET28a vector and expressed in *Escherichia coli* BL21-Star DE3. Bacterial cultures were grown in Luria–Bertani (LB) medium supplemented with 0.5 M isopropyl-β-d-1-thiogalactopyranoside (IPTG). Following cell lysis, the recombinant proteins were purified using affinity and ion exchange chromatography. Protein concentrations were determined via fluorimetry using the Qubit 2.0 system (Invitrogen Technologies, USA).

### Sample collection

**Fig. 1:**
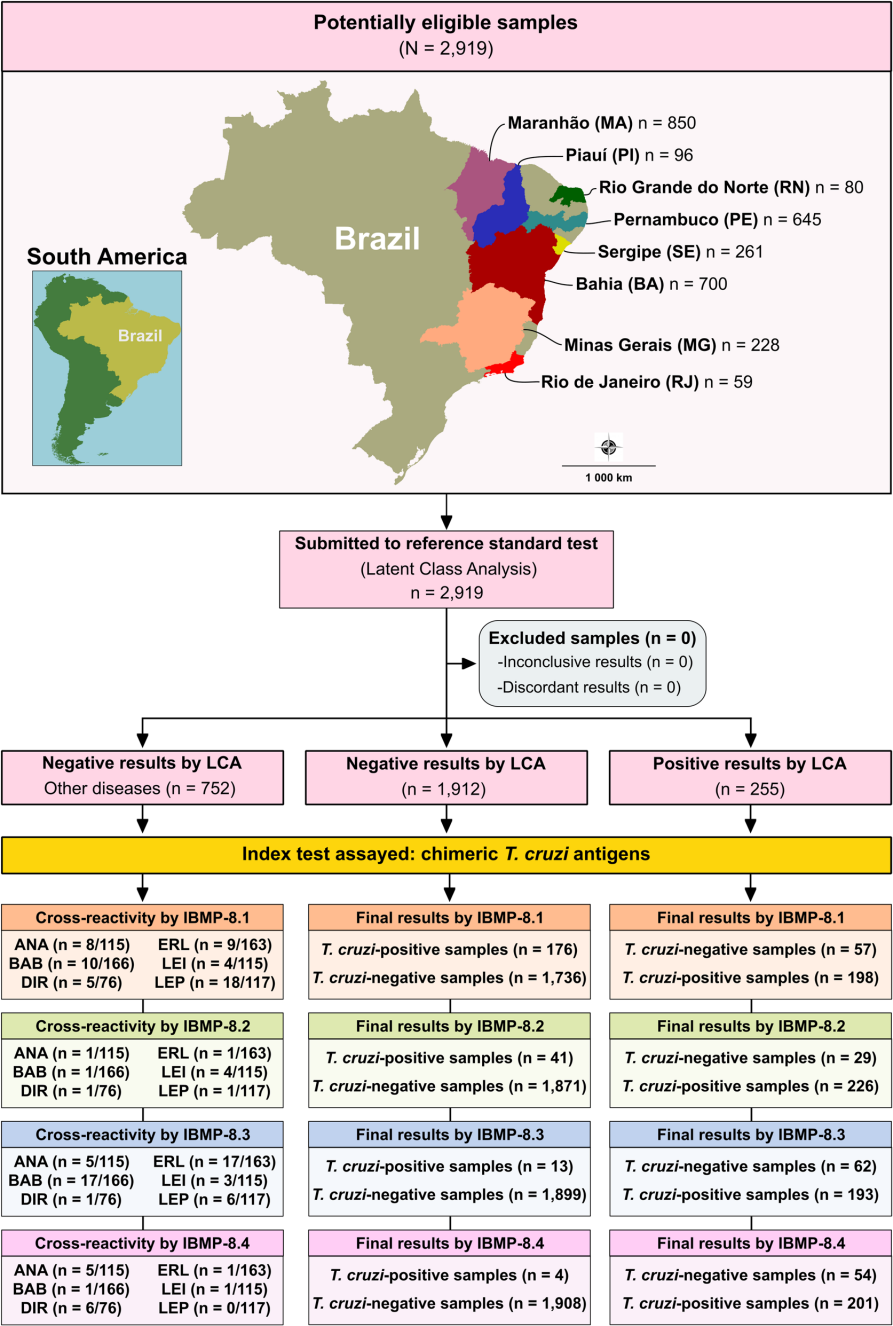
Flowchart of study design following the Standards for Reporting of Diagnostic Accuracy Studies (STARD) guidelines. The base map was obtained from the Brazilian Institute of Geography and Statistics (IBGE) and processed using QGIS v3.10 (http://qgis.osgeo.org). ANA, anaplasmosis; BAB, babesiosis; DIR, dirofilariasis, EHR, ehrlichiosis, LEI, leishmaniasis, LEP, leptospirosis, LCA , latent class analysis, *n*, number of samples

Sample size calculations were performed using OpenEpi [46], assuming 99% sensitivity and specificity, a 1.5% absolute error, and a 95% confidence level, resulting in a minimum requirement of 169 *T. cruzi*-positive and 169 *T. cruzi*-negative serum samples. In total, 2167 serum samples were collected from dogs across multiple endemic regions in Brazil, allowing for a robust evaluation of all four IBMP antigens via ELISA (Fig. 1). Samples originated from the following states: Bahia (BA; *n* = 566), Maranhão (MA; *n* = 850), Minas Gerais (MG; *n* = 220), Pernambuco (PE; *n* = 35), Piauí (PI; *n* = 96), Rio de Janeiro (RJ; *n* = 59), Rio Grande do Norte (RN; *n* = 80), and Sergipe (SE; *n* = 261). In addition, 752 serum samples from dogs with serologically or parasitologically confirmed infectious diseases unrelated to *T. cruzi* were included [47]. These comprised anaplasmosis (*n* = 115), babesiosis (*n* = 166), dirofilariasis (*n* = 76), ehrlichiosis (*n* = 163), visceral leishmaniasis (*n* = 115), and leptospirosis (*n* = 117). All samples, except for those with visceral leishmaniasis (17 from Bahia and 8 from Minas Gerais) and leptospirosis (all from Bahia), were collected in Pernambuco. All samples were tested using IBMP-based indirect ELISA (IBMP–ELISA), and results were analyzed using latent class analysis (LCA), as previously established by our group [48]. Samples were anonymized with unique codes to ensure operator blinding.

### IBMP–ELISA

Immunoassays were conducted as previously described by Leony et al. [44]. Flat-bottom 96-well polystyrene plates (Nunc Maxisorp^®^, USA) were coated with 25 ng/well IBMP antigen diluted in 100 µl 0.05 M carbonate/bicarbonate buffer (pH 9.6). Sensitization, blocking, and stabilization were performed simultaneously using a synthetic buffer (WellChampion^®^; Kem-En-Tec Diagnostics, Denmark) according to the manufacturer’s instructions. Serum samples, diluted 1:100 in phosphate-buffered saline (PBS; 0.05 M, pH 7.4), were incubated at 37 °C for 60 min. Subsequently, 100 µl horseradish peroxidase (HRP)-conjugated anti-dog IgG (Bio-Manguinhos, Fiocruz, Brazil) was added at a dilution of 1:20,000 for IBMP-8.3 and 1:40,000 for IBMP-8.1, IBMP-8.2, and IBMP-8.4. After a 30-min incubation at 37 °C and additional washes, TMB substrate (Kem-En-Tec) was added, and plates were incubated in the dark for 10 min. Reactions were stopped with 50 μl 0.3 M H_2_SO_4_, and absorbance was measured at 450 nm (SPECTRAmax 340PC^®^; Molecular Devices, USA), with background readings subtracted.

### Reference test

**Fig. 2:**
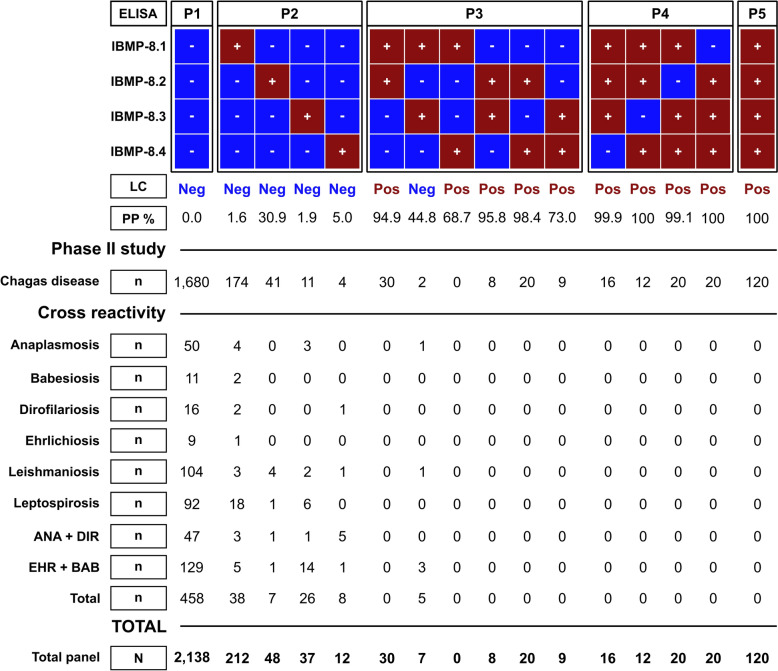
Latent class response patterns, a posteriori probability, and classification of canine serum samples tested with four chimeric *Trypanosoma cruzi* antigens for accurate diagnosis of Chagas disease. Samples are categorized as P1–P5 on the bases of the response patterns. Blue squares indicate negative results; red squares indicate positive results for a single IBMP chimeric antigen. LC, latent class; N, number of samples; Neg, negative; Pos, positive; PP, a posteriori probability of CD

In the absence of a definitive gold standard for canine CD diagnosis, a latent class analysis (LCA) model was employed to determine infection status, as described by Fontes et al. [48]. Samples were classified as positive if at least two IBMP chimeric antigens yielded positive results and the posterior probability (PP) exceeded 68%. Samples reactive exclusively to IBMP-8.1 and IBMP-8.3 with PP values below 50%, or those with no reactive antigens or only one positive result with PP < 31%, were considered negative (Fig. 2).

### Comparison with commercial ELISA

To benchmark the IBMP–ELISA against existing commercial assays, the Gold ELISA Chagas kit (REM Indústria e Comércio Ltda, Brazil), a human diagnostic kit previously adapted for canine use by our group [44], was employed. This kit incorporates recombinant antigens and lysates of epimastigotes from *T. cruzi* strains prevalent in Brazil. Immunoassays were conducted according to the manufacturer’s instructions, with minor adaptations. A total of 644 serum samples (154 positive, 490 negative) were analyzed. Samples were diluted 1:800 in sample dilution buffer, and 100 µl was added per well. Plates were incubated at 37 °C for 30 min, washed, and then incubated with 100 μl HRP-conjugated goat anti-dog IgG (1:40,000; Bio-Manguinhos, Fiocruz, Brazil) at 37 °C for 30 min. Detection was performed using the TMB Solution, and reactions were stopped with 50 μl of Stop Solution. Absorbance was measured at 450 nm using a SPECTRAmax 340PC (Molecular Devices, USA). In addition, 752 positive samples for unrelated diseases were used to evaluate cross-reactivity, including 115 positive for anaplasmosis, 166 for babesiosis, 76 for dirofilariasis, 163 for ehrlichiosis, 115 for visceral leishmaniasis, and 117 for leptospirosis.

### Statistical analysis

Data were analyzed using GraphPad Prism v9.5.1 (GraphPad Software, USA). Descriptive statistics included arithmetic and geometric means ± standard deviation, and coefficient of variation, as well as medians + interquartile ranges. Normality was assessed using the Shapiro–Wilk test, followed by Student’s *t* test or the Wilcoxon signed-rank test, as appropriate. Two-tailed *P* values < 0.05 were considered statistically significant. Cut-off values were determined using receiver operating characteristic (ROC) curves, with area under the curve (AUC) values interpreted as low (0.51–0.61), moderate (0.62–0.81), elevated (0.82–0.99), or outstanding (1.0) [49]. Results were expressed as a reactivity index (RI), defined as the ratio of the optical density (OD) of a sample to the cut-off. RI < 1.00 was considered negative, while RI ≥ 1.00 was considered positive. RI = 1.00 ± 10% was considered indeterminate (gray zone).

Diagnostic performance was evaluated using 2 × 2 contingency tables for sensitivity (Sen), specificity (Spe), accuracy (Acc), likelihood ratios (LR), diagnostic odds ratio (DOR), and pre- and post-test probabilities. To comprehensively assess the diagnostic accuracy of the four IBMP chimeric antigens, combinations of tests were analyzed using both serial and parallel strategies. In a parallel testing approach, multiple assays are performed simultaneously, and a positive result from any of them is sufficient to indicate infection. Conversely, in serial testing, subsequent assays are carried out on the basis of the outcome of the preceding one, and all tests must yield positive results to confirm the presence of disease [50]. Confidence intervals (95% CI) were calculated for all estimates. Agreement between LCA and IBMP–ELISA results was measured using Cohen’s kappa (*κ*) interpreted as: poor (*κ* ≤ 0), slight (0–0.20), fair (0.21–0.40), moderate (0.41–0.60), substantial (0.61–0.80), and almost perfect agreement (0.81–1.0) [51]. In compliance with Standards for Reporting of Diagnostic Accuracy Studies (STARD), a study flowchart (Fig. 1) and checklist (Supplementary Table 1) are provided [52].

## Results

### Latent class analysis

A total of 2919 canine sera were evaluated for *T. cruzi* infection using LCA to assess the diagnostic performance of four chimeric recombinant IBMP antigens. Of these, 2167 samples were included in the phase II study (individual RI values are presented in Supplementary Table 2), while 752 samples were reserved for cross-reactivity assessment (individual RI values are presented in Supplementary Table 3). Within the phase II cohort, LCA identified 1912 samples as negative (88.2%) and 255 as positive for *T. cruzi* antibodies (11.8%). Among the LCA-negative samples, 87.9% (1680/1912) tested negative for all four chimeric antigens by ELISA (Fig. 2). LCA-negative samples yielding a positive result for only one antigen were observed in 230 cases (12%; 230/1912), distributed as follows: 174 (75.7%) for IBMP-8.1, 41 (17.8%) for IBMP-8.2, 11 (4.8%) for IBMP-8.3, and four (1.7%) for IBMP-8.4. The a posteriori probability of positivity for these samples was less than 31%, supporting their accurate classification as negative (Fig. 2). Only one negative sample was positive for both IBMP-8.1 and IBMP-8.3, with a posteriori probability below 45%.

Among LCA-positive samples, 120 of 255 (47%) were positive for all IBMP antigens, while 67 (26.3%) were positive for two antigens: 30 (44.8%) for IBMP-8.1 + IBMP-8.2, eight (11.9%) for IBMP-8.2 + IBMP-8.3, 20 (29.9%) for IBMP-8.2 + IBMP-8.4, and nine (13.4%) for IBMP-8.3 + IBMP-8.4. The probability of positivity in these samples exceeded 68.7%, indicating high confidence in classification. In total, 68 samples (26.7%) were positive for three antigens, with the following distributions: 16 (23.5%) for IBMP-8.1 + IBMP-8.2 + IBMP-8.3, 12 (17.7%) for IBMP-8.1 + IBMP-8.2 + IBMP-8.4, 20 (29.4%) for IBMP-8.1 + IBMP-8.3 + IBMP-8.4, and 20 (29.4%) for IBMP-8.2 + IBMP-8.3 + IBMP-8.4, all with posterior probabilities above 99.1% (Fig. 2).

When each IBMP antigen was analyzed individually: IBMP-8.1 classified 1793 samples (82.7%) as negative and 374 (17.3%) as positive; IBMP-8.2 classified 1900 (87.7%) as negative and 267 (12.3%) as positive; IBMP-8.3 classified 1961 (90.5%) as negative and 206 (9.5%) as positive; and IBMP-8.4 classified 1962 (90.5%) as negative and 205 (9.5%) as positive. Discordant results were observed in 233 samples (10.8%) for IBMP-8.1, 70 (3.2%) for IBMP-8.2, 75 (3.5%) for IBMP-8.3, and 58 (2.7%) for IBMP-8.4. Each IBMP antigen was evaluated separately:

*IBMP-8.1 antigen*: identified 57 of 255 LCA-positive samples (19.1%) as negative and 176 of 1912 LCA-negative samples (9.2%) as positive. The probability of positivity for these false negatives ranged from 73 to ≥ 95.8%. Most false positives (98.9%) had a near-zero probability of positivity (1.6%), with only two samples showing a probability of 44.8%.

*IBMP-8.2 antigen*: identified 29 of 255 LCA-positive samples (14.4%) as negative and 41 of 1912 LCA-negative samples (2.1%) as positive. All false positives had a 30.9% probability of positivity.

*IBMP-8.3 antigen*: identified 62 of 255 LCA-positive samples (24.3%) as negative and 13 of 1912 LCA-negative samples (0.7%) as positive. Most false-positives (69.2%) had near-zero probability (1.9%) of positivity.

*IBMP-8.4 antigen*: classified 54 of 255 LCA-positive samples (21.2%) as negative and four of 1912 LCA-negative samples (0.21%) as positive. All false positives had a 5% probability of being positive.

All 752 sera used for cross-reactivity evaluation were classified as negative by LCA. Most (96.3%) showed a near-zero probability of positivity (1.9%); the remainder ranged from 5% to 44.8%.

### Individual IBMP–ELISA performance

Using LCA status as the reference, the area under the ROC curve (AUC) ranged from 90.5% for IBMP-8.1 to 98.0% for IBMP-8.2 and IBMP-8.4, and 98.2% for IBMP-8.3, indicating high overall diagnostic capacity. The AUC for IBMP-8.1 was significantly lower than that for the other antigens, as indicated by nonoverlapping 95% confidence intervals.

For *T. cruzi*-positive sera, IBMP-8.2 yielded the highest IgG levels (RI = 1.43; interquartile range (IQR) 1.17–1.91), while IBMP-8.4 had the lowest (RI = 1.28; IQR 1.02–1.85); these differences were not statistically significant. Similarly, no significant differences were observed between IBMP-8.1 (RI = 1.31; IQR 1.03–1.88) and IBMP-8.3 (RI = 1.31; IQR 1.00–1.81).

**Fig. 3:**
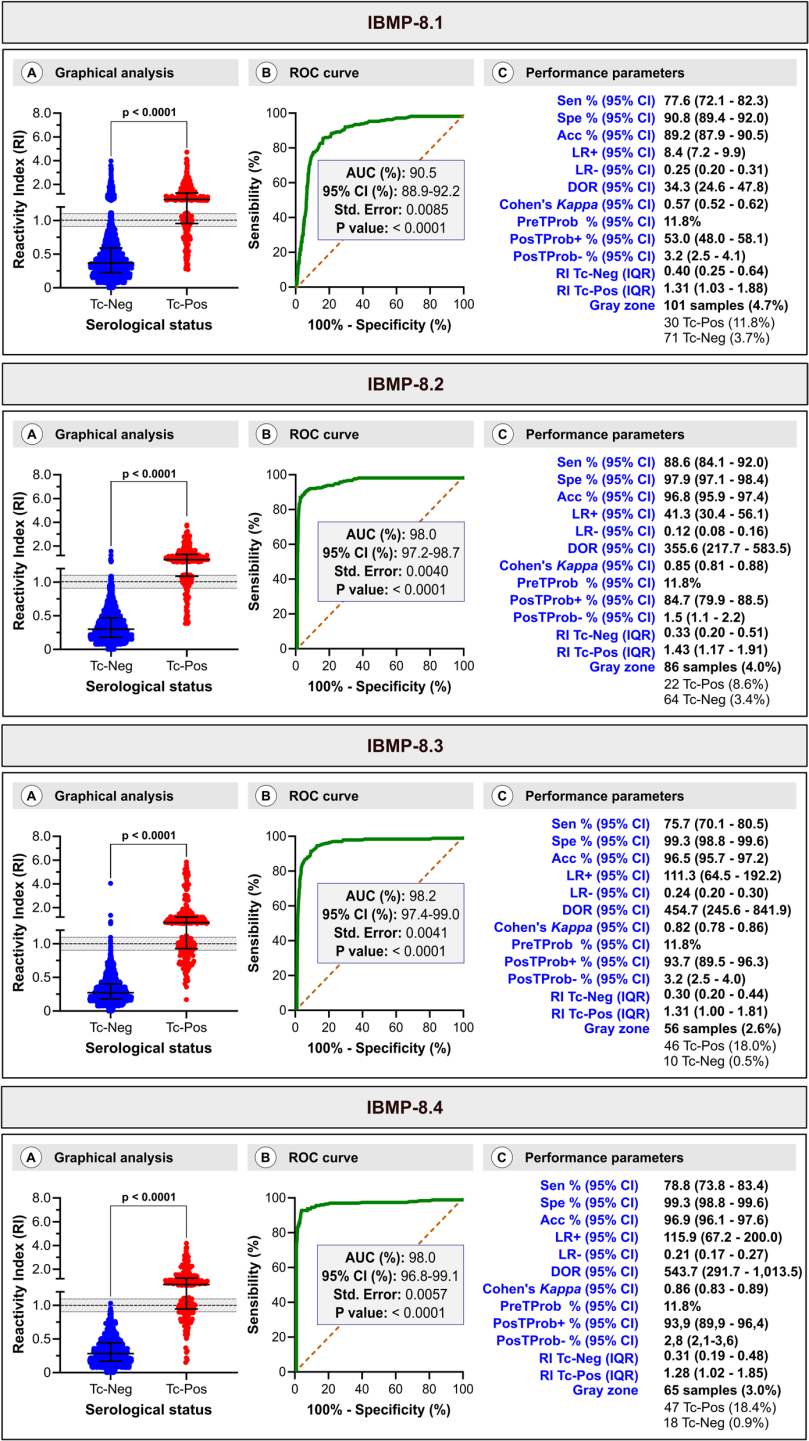
Performance evaluation of chimeric recombinant *Trypanosoma cruzi* antigens using ELISA. **A** Reactivity index (RI) for each antigen tested against 255 *T. cruzi*-positive and 1912 *T. cruzi*-negative samples. The cutoff is 1.0; the shaded area denotes the gray zone. Horizontal lines represent medians (IQR). **B** ROC curves and AUC for each IBMP antigen. **C** Diagnostic performance parameters. Acc, accuracy; AUC, area under the ROC curve; DOR, diagnostic odds ratio; LR, likelihood ratio; PosTProb, posttest probability; PreTProb, pretest probability; Sen, sensitivity; Spe, specificity; Tc-Neg, *T. cruzi*-negative; Tc-Pos, *T. cruzi*-positive

Among 255 *T. cruzi*-positive samples, IBMP-8.2 demonstrated the highest sensitivity (88.6%), with 29 false negatives; nine of these were also false negatives for IBMP-8.1. Other antigens showed sensitivities of 77.6% (IBMP-8.1), 75.7% (IBMP-8.3), and 78.8% (IBMP-8.4). IBMP-8.2 was significantly more sensitive than the others. For *T. cruzi*-negative samples, IBMP-8.3 and IBMP-8.4 exhibited specificities ≥ 99.0%, surpassing those of IBMP-8.1 and IBMP-8.2, which had more false positives (specificities: 90.8% with 176 false positives and 97.9% with 41 false positives), respectively. IBMP-8.1 was significantly less specific than IBMP-8.2. For negative samples, IBMP-8.3 and IBMP-8.4 produced the lowest IgG levels (RI = 0.30 and 0.31, respectively), while IBMP-8.1 had the highest (RI = 0.40), with no significant difference among antigens (Fig. 3).

Considering RI values of 1.0 ± 0.10 as the gray zone, only ten *T. cruzi*-negative samples (0.5%) were inconclusive with IBMP-8.3, while 18 (0.9%), 64 (3.4%), and 71 (3.7%) were inconclusive with IBMP-8.4, IBMP-8.2, and IBMP-8.1, respectively. Among the *T. cruzi*-positive samples, the gray zone included 22 (8.6%) for IBMP-8.2, 30 (11.8%) for IBMP-8.1, 46 (18.0%) for IBMP-8.3, and 47 (18.4%) for IBMP-8.4. Overall, 2.6% (56/2167) of samples tested with IBMP-8.3, 3.0% (65/2167) with IBMP-8.4, 4.0% (86/2167) with IBMP-8.2, and 4.7% (101/2167) with IBMP-8.1 had RI values within the gray zone.

The IBMP antigens exhibited accuracies of 96.9% (IBMP-8.4), 96.8% (IBMP-8.2), and 96.5% (IBMP-8.3). Owing to the higher false-negative and false-positive rates, the accuracy of IBMP-8.1 was lower (89.2%). As shown in Fig. 3, IBMP-8.4 presented the highest DOR (543.7), followed by IBMP-8.3 (454.7), IBMP-8.2 (355.6), and IBMP-8.1 (34.3). Cohen’s kappa indicated moderate agreement for IBMP-8.1 (*κ* = 0.57) and almost perfect for IBMP-8.3 (*κ* = 0.82), IBMP-8.2 (*κ* = 0.85), and IBMP-8.4 (*κ* = 0.86). IBMP-8.2, IBMP-8.3, and IBMP-8.4 demonstrated superior performance by ROC and DOR analyses, while IBMP-8.1 had the lowest performance. Importantly, IBMP-8.2 had the highest sensitivity, while IBMP-8.3 and IBMP-8.4 had the highest specificity.

### Antigen sensitivity by geographical origin

**Fig. 4:**
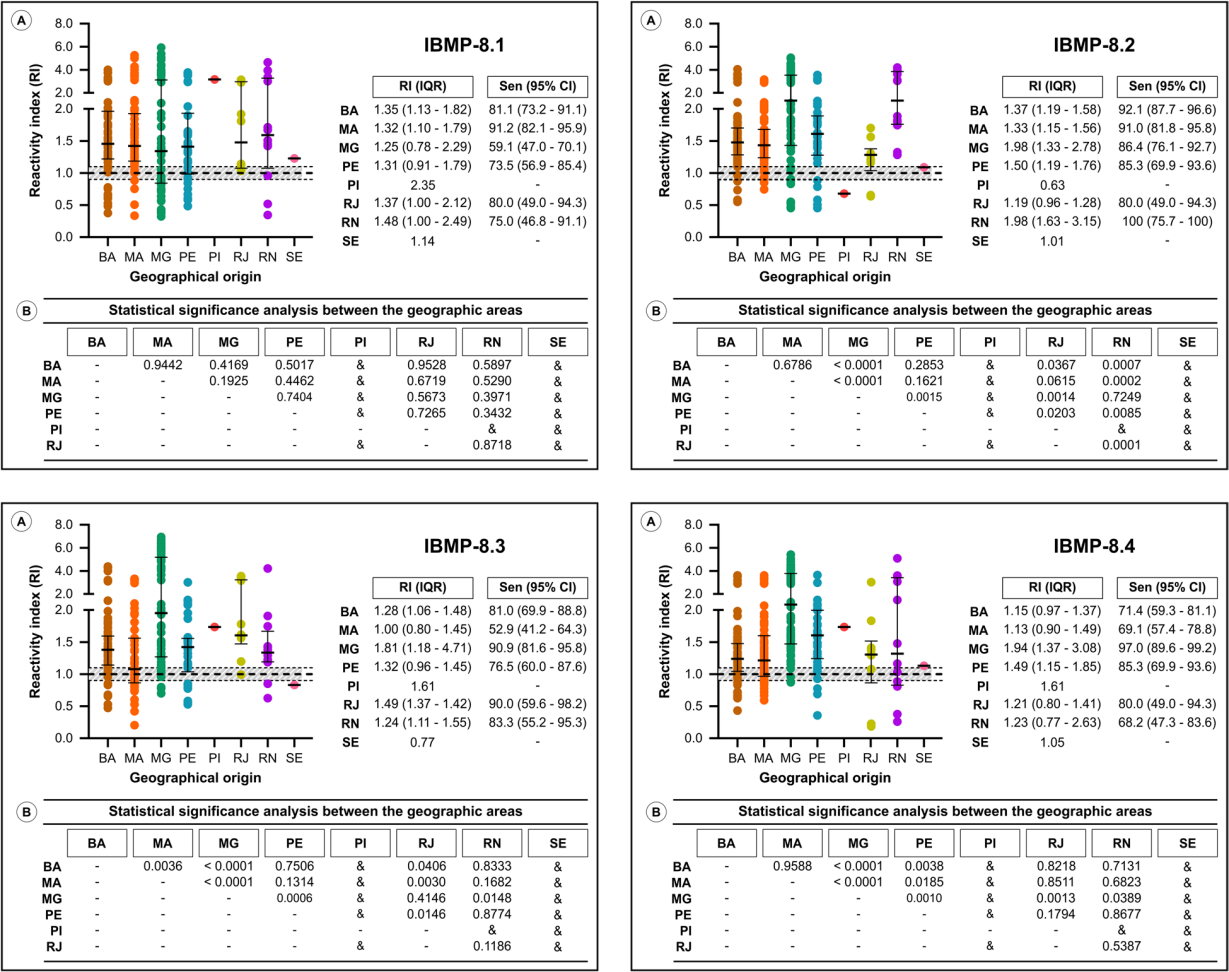
ELISA performance employing chimeric recombinant *Trypanosoma cruzi* antigens in sera from different Brazilian regions. **A** Reactivity index and sensitivity for *T. cruzi*-positive samples by region. The cutoff is 1.0; the shaded area represents the gray zone (RI = 1.0 ± 0.10). Horizontal lines represent medians (IQR). **B** Statistical analysis of the RI signal between regions. BA, Bahia; MA, Maranhão; MG, Minas Gerais; PE, Pernambuco; PI, Piauí; RJ, Rio de Janeiro; RN, Rio Grande do Norte; SE, Sergipe; Sen, sensitivity; CI, confidence interval; &, analysis not performed owing to small sample size (*n* = 1)

To account for *T. cruzi* genetic diversity, positive samples were stratified by geographic origin to assess humoral responses to IBMP antigens in infected dogs from different Brazilian regions (Fig. 4; individual RI values are presented in Supplementary Table 2). IBMP-8.1 was recognized uniformly across all regions. In contrast, the signals from IBMP-8.2, IBMP-8.3, and IBMP-8.4 varied significantly by region. The lowest sensitivity was observed for Maranhão (MA) with IBMP-8.3 (52.9%) and IBMP-8.4 (69.1%), whereas IBMP-8.2 achieved a sensitivity of 91% in this state. Sensitivity below 80% was also observed for IBMP-8.1 in MG, PE, RJ, and RN; for IBMP-8.3 in PE; and for IBMP-8.4 in BA and RN. Sensitivity could not be determined for PI and SE owing to the presence of a single positive sample in each.

### IBMP–ELISA performance: serial and parallel approaches

To minimize diagnostic uncertainty, serial and parallel testing strategies were employed in conjunction with the IBMP–ELISA results (Table 1). Parallel analysis consistently increased sensitivity compared with individual or serial approaches, with the IBMP-8.1 + IBMP-8.3 pair yielding the lowest combined sensitivity (94.6%). Specificity was ≥ 90% for individual IBMP-8.1, IBMP-8.2, or IBMP-8.4, and for any combination thereof, except for IBMP-8.1 + IBMP-8.2 (88.9%). Combinations including IBMP-8.3 in parallel achieved 90% specificity. Serial combinations generally did not improve accuracy over individual tests, except for IBMP-8.1 alone or in combination (89.2–90.8%). In parallel, the combinations of IBMP-8.2 + IBMP-8.3, IBMP-8.2 + IBMP-8.4, and IBMP-8.3 + IBMP-8.4 provided the highest accuracy, outperforming IBMP-8.1 alone but not the other antigens.

**Table 1:** Diagnostic performance of IBMP–ELISA, individually and in combination, using serial and parallel approaches

IBMP–ELISA	Approach	Sen (95% CI)	Spe (95% CI)	Acc (95% CI)
IBMP-8.1	Individual	77.6 (72.1–82.3)	90.8 (89.4–92.0)	89.2 (87.9–90.5)
IBMP-8.2	Individual	88.6 (84.1–92.0)	97.9 (97.1–98.4)	96.8 (95.9–97.4)
IBMP-8.3	Individual	75.7 (70.1–80.5)	99.3 (98.8–99.6)	96.5 (95.7–97.2)
IBMP-8.4	Individual	78.8 (73.8–83.4)	99.3 (98.8–99.6)	96.9 (96.1–97.6)
IBMP-8.1 + IBMP-8.2	Serial	68.8 (60.6–75.7)	99.8 (99.7–99.9)	96.1 (95.1–97.0)
	Parallel	97.4 (95.6–98.6)	88.9 (86.8–90.5)	89.9 (87.8–91.5)
IBMP-8.1 + IBMP-8.3	Serial	58.7 (50.5–66.3)	99.9 (99.9–100)	95.1 (94.1–96.0)
	Parallel	94.6 (91.7–96.5)	90.2 (88.3–91.6)	90.7 (88.7–92.2)
IBMP-8.1 + IBMP-8.4	Serial	61.1 (53.2–68.5)	99.9 (99.9–100)	95.4 (94.4–96.3)
	Parallel	95.3 (92.7–97.1)	90.2 (88.3–91.6)	90.8 (88.8–92.3)
IBMP-8.2 + IBMP-8.3	Serial	67.1 (59.0–74.1)	100 (99.9–100)	96.1 (95.1–96.9)
	Parallel	97.2 (95.2–98.4)	97.2 (95.9–98.0)	97.2 (95.9–98.1)
IBMP-8.2 + IBMP-8.4	Serial	69.8 (62.1–76.7)	100 (99.9–100)	96.4 (95.5–97.2)
	Parallel	97.6 (95.8–98.7)	97.2 (95.9–98.0)	97.3 (95.9–98.1)
IBMP-8.3 + IBMP-8.4	Serial	59.7 (51.7–67.1)	100 (99.9–100)	95.2 (94.3–96.1)
	Parallel	94.8 (92.2–96.8)	98.6 (97.6–99.2)	98.2 (97.0–98.9)

Sen, sensitivity; Spe, specificity; Acc, accuracy; CI, confidence interval

### Cross-reactivity analysis

**Fig. 5:**
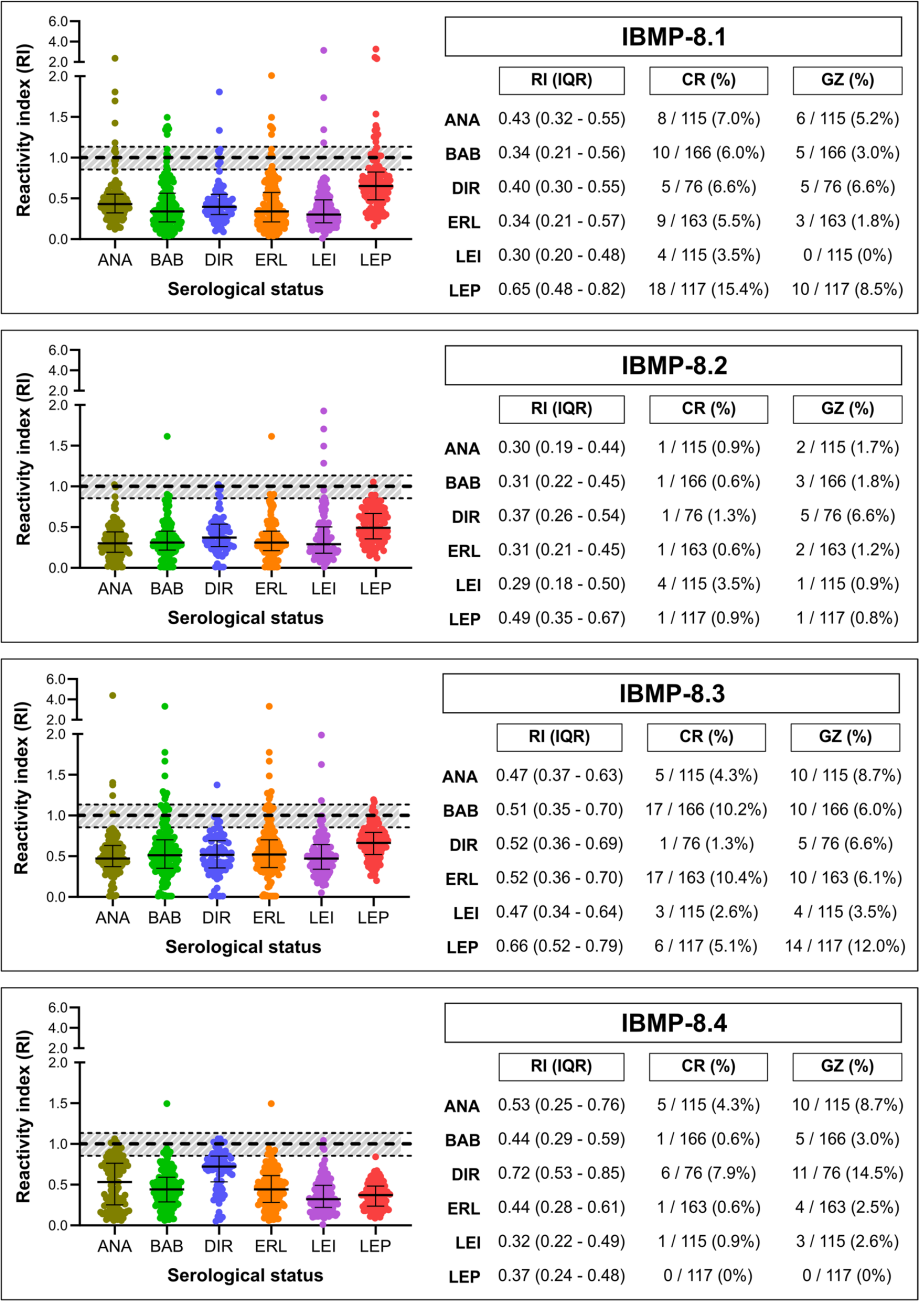
Cross-reactivity analysis of IBMP chimeric proteins with sera from dogs with unrelated diseases. The cutoff is 1.0; the shaded area is the gray zone (RI = 1.0 ± 0.10). Horizontal lines represent geometric means. ANA, anaplasmosis; BAB, babesiosis; CR, cross-reactivity; DIR, dirofilariasis; ERL, ehrlichiosis; GZ, gray zone; IQR, interquartile range; LEI, leishmaniasis; LEP, leptospirosis; RI, reactivity index

Cross-reactivity (RI ≥ 1.0) was assessed using 752 sera from dogs with unrelated diseases (Fig. 5; individual RI values are presented in Supplementary Table 3). Frequencies were 7.2% (*n* = 54) for IBMP-8.1, 1.2% (*n* = 9) for IBMP-8.2, 6.5% (*n* = 49) for IBMP-8.3, and 1.9% (*n* = 14) for IBMP-8.4. The proportion of inconclusive results for IBMP-8.2 (1.8%; *n* = 14) was lower than for IBMP-8.1 (3.9%), IBMP-8.4 (4.3%), and IBMP-8.3 (5.9%). Among *Leishmania* spp. samples, only one cross-reacted with IBMP-8.4, three with IBMP-8.3, and four each with IBMP-8.1 and IBMP-8.2.

### Comparison with canine-adapted commercial human *T. cruzi* ELISA

**Fig. 6:**
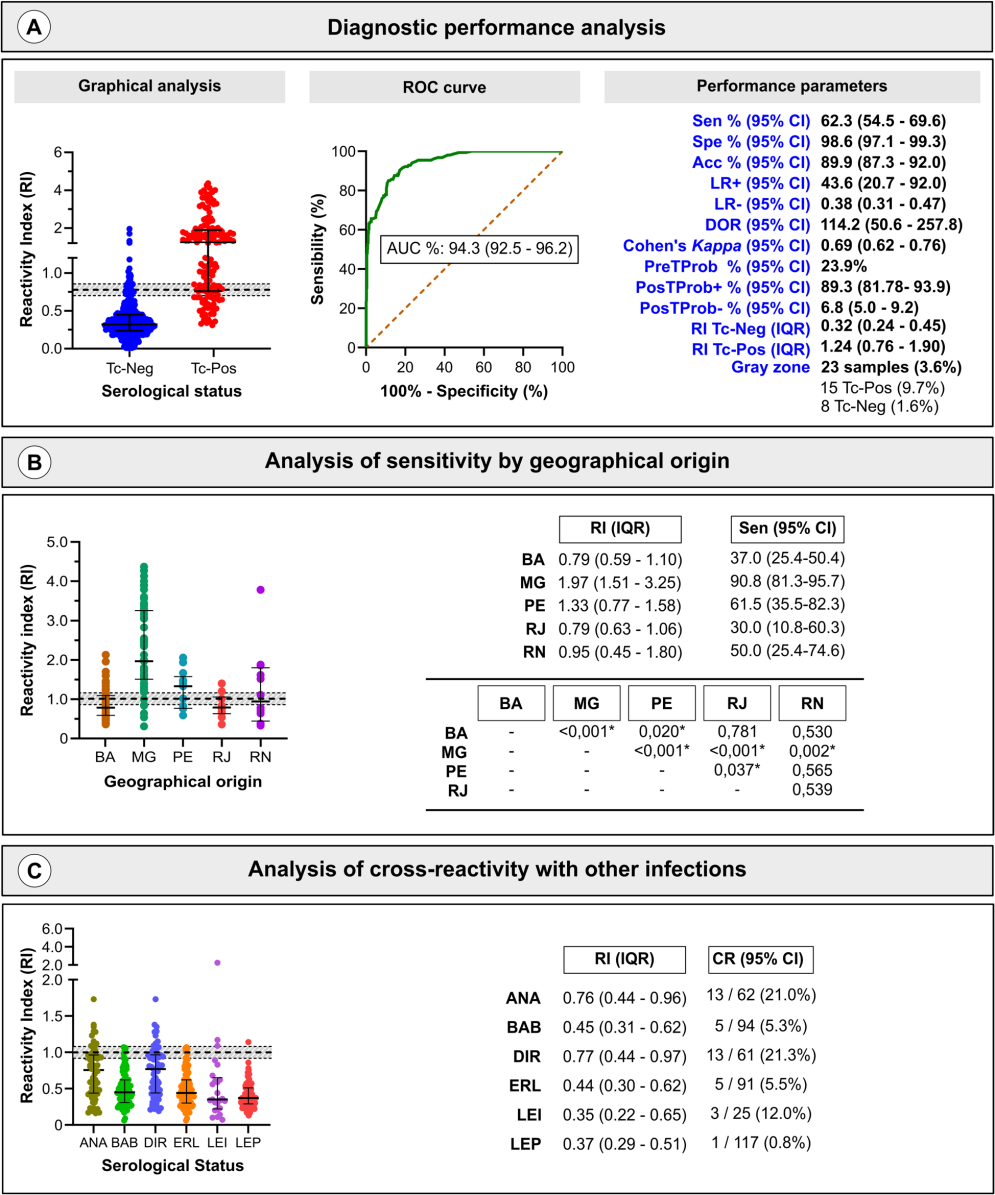
Performance of a commercial human-adapted *T. cruzi* ELISA (Gold ELISA Chagas) for canine Chagas disease diagnosis. **A** RI for the commercial kit against 154 *T. cruzi*-positive and 490 *T. cruzi*-negative samples. Middle: ROC curve and AUC. Right: performance parameters. **B** Performance by region. **C** Cross-reactivity with sera from dogs with unrelated diseases. The cutoff is 1.0; the shaded area is the gray zone. Horizontal lines and numbers represent geometric means (95% CIs). Acc, accuracy; ANA, anaplasmosis; AUC, area under the ROC curve; BA, Bahia; BAB, babesiosis; CI, confidence interval; DIR, dirofilariasis; DOR, diagnostic odds ratio; ERL, ehrlichiosis; LEI, leishmaniasis; LEP, leptospirosis; LR, likelihood ratio; MG, Minas Gerais; PE, Pernambuco; PosTProb, posttest probability; PreTProb, pretest probability; RI, reactivity index; RJ, Rio de Janeiro; RN, Rio Grande do Norte; Sen, sensitivity; Spe, specificity; Tc-Neg, *T. cruzi*-negative; Tc-Pos, *T. cruzi*-positive

To benchmark IBMP–ELISAs, 154 *T. cruzi*-positive and 490 *T. cruzi*-negative canine sera were tested with the Gold ELISA Chagas kit. The commercial test showed a sensitivity of 62.3% (95% CI: 54.5–69.6%) (Fig. 6; individual RI values are presented in Supplementary Table 4). This was comparable to IBMP-8.1 and IBMP-8.3 (overlapping 95% CIs), but IBMP-8.2 and IBMP-8.4 were significantly more sensitive. Specificity for the Gold ELISA Chagas kit was 98.6% (95% CI: 97.1–99.3%), similar to IBMP-8.2, IBMP-8.3, and IBMP-8.4 but higher than IBMP-8.1. DOR analysis indicated that the Gold ELISA Chagas kit and IBMP-8.3/IBMP-8.2 had similar diagnostic performance, while IBMP-8.4 outperformed and IBMP-8.1 underperformed relative to the commercial kit. The commercial kit’s signal varied significantly by region. Cross-reactivity was 8.9% (40/450) for the commercial kit, compared with 7.2% for IBMP-8.1, 0.9% for IBMP-8.2, 6.5% for IBMP-8.3, and 1.9% for IBMP-8.4. Among *Leishmania* spp. samples, three cross-reacted with the commercial kit (Fig. 6).

### Sensitivity versus specificity comparison

**Fig. 7:**
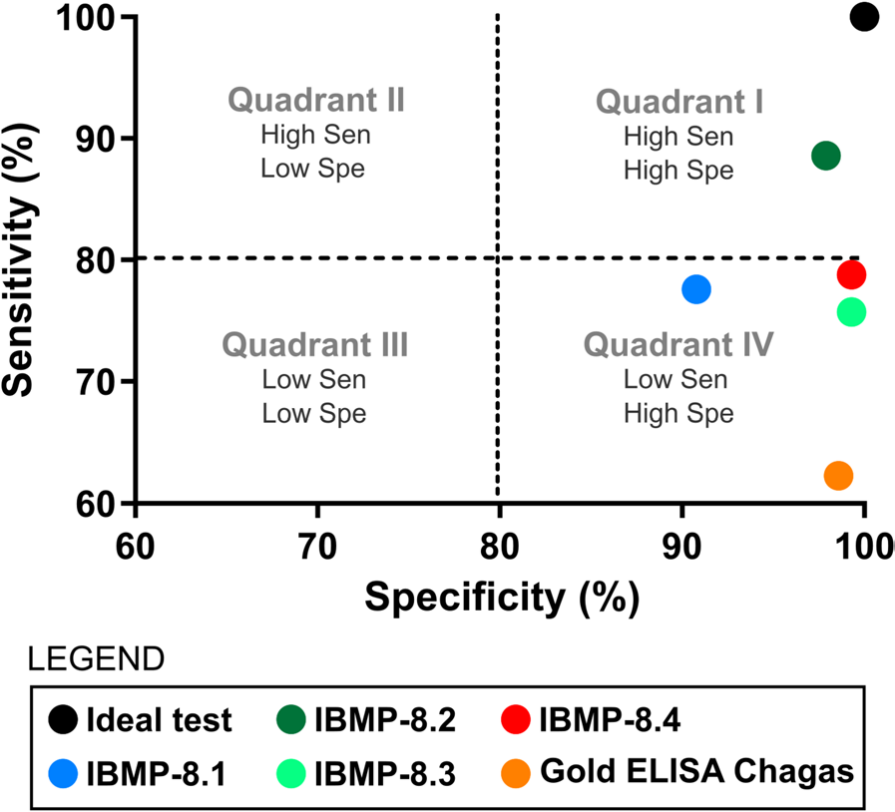
Sensitivity versus specificity for IBMP chimeras and a commercial *T. cruzi* ELISA. The black dot represents an ideal diagnostic assay (100% accuracy). Sen, sensitivity; Spe, specificity

Figure 7 shows the relationship between sensitivity and specificity for IBMP antigens and the commercial ELISA. The ideal assay (black dot) is 100% accurate. IBMP-8.2 exhibited the best overall performance, followed by IBMP-8.4, IBMP-8.3, IBMP-8.1, and the Gold ELISA Chagas kit. IBMP-8.2 achieved high sensitivity and specificity (quadrant I), while IBMP-8.1, IBMP-8.3, IBMP-8.4, and the commercial kit showed high specificity but lower sensitivity (quadrant IV). No assays fell into quadrants II (high sensitivity, low specificity) or III (low sensitivity, low specificity).

## Discussion

Despite canines’ pivotal role in sustaining the domestic transmission cycle of CD and the clinical similarities between canine and human *T. cruzi* infections, few commercial serological assays are currently available for anti-*T. cruzi* antibody detection in dogs. Consequently, research on canine CD has relied on in-house or adapted human commercial assays [53–57]. In this study, we comprehensively evaluated the diagnostic performance of four chimeric recombinant *T. cruzi* antigens (IBMP-8.1, IBMP-8.2, IBMP-8.3, and IBMP-8.4) for the detection of anti-*T. cruzi* antibodies in sera from naturally infected dogs across diverse endemic and nonendemic regions of Brazil.

All IBMP proteins demonstrated high discriminatory power between *T. cruzi*-positive and negative samples, with area under the ROC curve (AUC) values ranging from 89.0% to 97.4%. Notably, IBMP-8.4 exhibited the highest AUC, consistent with previous phase I findings (AUC = 100%) [44]. In human studies, all four antigens have consistently achieved AUC values above 99% [26, 35, 39–42]. When compared with a commercial human-adapted test (Gold ELISA Chagas), IBMP-8.2, IBMP-8.3, and IBMP-8.4 outperformed the commercial assay in terms of AUC, whereas IBMP-8.1 showed a lower value; although not statistically significant.

Among the chimeric antigens, IBMP-8.2 displayed the highest diagnostic sensitivity, exceeding that of the other IBMP proteins and the Gold ELISA Chagas assay. While IBMP-8.1 and IBMP-8.3 exhibited sensitivities similar to those of the commercial kit, only IBMP-8.2 achieved a sensitivity greater than 90%. Interestingly, these results diverge from those of the phase I study, where IBMP-8.3 was the most sensitive antigen [44]. This discrepancy likely reflects the broader geographic and genetic diversity of *T. cruzi* strains included in the present study, highlighting the impact of parasite heterogeneity on serological assay performance. *T. cruzi* is classified into seven discrete typing units (DTUs), with further regional subtypes (clonets) [58–60], and this diversity can influence antigen recognition. In addition, differences in the amino acid composition of IBMP antigens may limit their reactivity with antibodies elicited by certain circulating strains. Stratification of positive samples by geographic origin confirmed that humoral responses to IBMP antigens vary regionally. To enhance detection rates, we evaluated serial and parallel combinations of IBMP antigens. Parallel testing, particularly combinations involving IBMP-8.2 with IBMP-8.4 or IBMP-8.1, yielded sensitivities exceeding 97%, supporting the utility of multiantigen approaches for screening purposes.

Regarding *T. cruzi*-negative samples, specificity values ranged from 90.6% to 99.6%, with IBMP-8.3 and IBMP-8.4 providing the highest specificities (≥ 99.0%). These findings align with phase I data, where IBMP-8.3 was the most specific antigen [44]. Parallel testing with IBMP-8.3 and IBMP-8.4 achieved a specificity above 98%, while serial combinations yielded specificities ranging from 99.7% to 100%, regardless of antigen pairing. Thus, both parallel and serial approaches, especially IBMP-8.3 + IBMP-8.4, may be valuable for confirmatory diagnostics. Accuracy was comparable for IBMP-8.2, IBMP-8.3, and IBMP-8.4 but lower for IBMP-8.1 owing to a higher rate of misclassification. In contrast, for human CD diagnosis, IBMP-8.1 and IBMP-8.4 have demonstrated superior performance [26, 39–42], and these antigens are included in the TR Chagas lateral flow immunochromatographic assay (Bio-Manguinhos, Fiocruz-RJ, Brazil), which achieves 100% accuracy in humans [39, 61]. The reduced performance of IBMP-8.1 in dogs may be a reflection in differences on antigen processing and presentation or differences in immunoglobulin VH and VL repertoires between canine breeds and humans [62]. Therefore, IBMP-8.1 alone is not recommended for canine CD diagnosis unless incorporated within a latent class model.

While sensitivity, specificity, and accuracy are essential metrics, they do not fully capture the clinical utility of a diagnostic test. Likelihood ratios (LR) and diagnostic odds ratios (DOR) provide more direct measures of a test’s impact on clinical decision-making. In this study, IBMP-8.4 achieved a positive LR of 250.9, indicating that a *T. cruzi*-infected dog is approximately 251 times more likely to be correctly diagnosed using this antigen. The DOR for IBMP-8.4 (1007.6) surpassed those for IBMP-8.3 (270.9), IBMP-8.2 (257.1), and IBMP-8.1 (28.6). All IBMP antigens except IBMP-8.1 outperformed the Gold ELISA Chagas kit (DOR = 114.2). Importantly, LR and DOR are robust metrics in phase II studies, as they are independent of disease prevalence.

Cross-reactivity analysis revealed limited seropositivity with unrelated diseases, consistent with the design of IBMP antigens, which are composed of *T. cruzi*-specific fragments. BLASTp analysis against the NCBI database confirmed minimal sequence similarity with nonpathogenic canine and human microorganisms [41]. IBMP-8.1 and IBMP-8.3 exhibited the highest cross-reactivity rates (7.2% and 6.5%, respectively), whereas IBMP-8.2 and IBMP-8.4 showed minimal cross-reactivity (0.9% and 1.9%, respectively), supporting their use in co-endemic settings. Anti-*Leishmania* spp. antibodies are a well-documented source of cross-reactivity in CD serology [22, 23, 34, 63–65]. In this study, cross-reactivity with *Leishmania* spp. ranged from 0.9% to 3.5%, higher than observed in phase I, where no cross-reactivity was detected for IBMP-8.1, IBMP-8.2, and IBMP-8.4. Other pathogens, such as *Babesia*, *Ehrlichia*, and *Leptospira*, also induced cross-reactivity with IBMP-8.1 and IBMP-8.3 but not with IBMP-8.2 and IBMP-8.4. Overall, cross-reactivity rates were low, particularly for IBMP-8.2, reinforcing the suitability of IBMP chimeras for use in regions of co-endemicity.

The principal limitation of this study was the absence of a validated reference standard for sera characterization. To address this, we employed a reference array of chimeric *T. cruzi* antigens and latent class analysis (LCA) as a surrogate gold standard, which provided robust diagnostic precision in the absence of a true reference. Additional limitations include the restricted geographic sampling, potential under representation of certain DTUs, and the lack of samples positive for other *Trypanosoma* species (e.g., *T. caninum*, *T. evansi*, and *T. rangeli*). Future studies should incorporate broader sampling from additional Brazilian states, especially the North region, and from other Latin American countries where *T. cruzi* is endemic. Despite these limitations, our findings confirm the remarkable diagnostic performance of IBMP chimeric antigens for chronic CD in dogs, with IBMP-8.2 and IBMP-8.4 demonstrating the highest accuracy.

## Conclusions

Our results demonstrate that these four chimeric recombinant *T. cruzi* IBMP antigens reliably discriminate between positive and negative canine sera. The accuracy of IBMP-8.2, IBMP-8.3, and IBMP-8.4 was consistent across geographic regions, supporting their potential use in commercial diagnostic kits. Accordingly, a lateral flow immunochromatographic assay incorporating two distinct IBMP antigens could be effectively deployed for *T. cruzi* transmission surveillance in endemic areas and veterinary diagnostic applications.

## Supplementary Information

**Additional file 1. S1 Table:** STARD checklist. Standards for the Reporting of Diagnostic Accuracy Studies. (STARD) checklist for reporting of studies of diagnostic accuracy.

**Additional file 2. S2 Table:** Reactivity Index for diagnostic performance assessment.

**Additional file 3. S3 Table:** Reactivity Index for cross-reactivity assessment.

**Additional file 4. S4 Table:** Reactivity Index for comparison with commercial *T. cruzi* ELISA.

## Data Availability

Data supporting the main conclusions of this study are included in the manuscript.

## Abbreviations

Acc: Accuracy
AUC: Area under the curve
BA: Bahia
CD: Chagas disease
CI: Confidence interval
DOR: Diagnostic odds ratio
ELISA: Enzyme-linked immunosorbent assay
HRP: Horseradish peroxidase
IBMP: Instituto de Biologia Molecular do Paraná
IPTG: Isopropyl-β-d-1-thiogalactopyranoside
IQR: Interquartile range
LB: Lysogenic broth
LCA: Latent class analysis
LR: Likelihood ratio
MA: Maranhão
MG: Minas Gerais
OD: Optical density
PBS: Phosphate-buffered saline
PE: Pernambuco
PI: Piauí
PP: Posterior probability
RI: Reactivity index
RJ: Rio de Janeiro
RN: Rio Grande do Norte
ROC: Receiver operating characteristic
SE: Sergipe
Sen: Sensitivity
Spe: Specificity
STARD: Standards for Reporting of Diagnostic Accuracy Studies
TBM: 3,3′,5,5′-tetramethylbenzidine
WHO: World Health Organization
